# Supplementary material for: Jointly Optimized Spatial Histogram UNET Architecture (JOSHUA) for Adipose Tissue Segmentation
Source: BME Front. 2022 Jun 3;2022:9854084. doi: 10.34133/2022/9854084 (PMC10521712; doi:10.34133/2022/9854084)
Supplement: Supplementary Materials — Figure S1: example images from each histological dataset for adipose tissue (S1a) and cancer gland segmentation (S1b). Figure S2: color histograms (16 bins, equally spaced) of the normalized intensity values of each image in the SFBHI dataset (excludes test images). Figure S3: color histograms (16 bins, equally spaced) of the normalized intensity values of each image in the GlaS dataset (excludes test images). Figure S4: example segmentation results on adipose-poor samples (i.e., less than 1% of adipose pixels in image) from each model on the SFBHI dataset. Table S1: metrics for each data split on validation images in SFHBI dataset trained on UNET with binary cross entropy. Table S2: global distribution of random data split based on time. Table S3: global distribution of random data split based on conditions. Table S4: global distribution of stratified 5-fold time data split. Table S5: global distribution of stratified 5-fold condition data split. Table S6: global distribution of 4-fold with time data split. Table S7: global distribution of validate on week 8 data split. Table S8: metrics for each model on images in SFHBI dataset Table S9: metrics for each model on images in GlaS dataset trained with binary cross entropy loss. [file 9854084.f1.zip › PeeplesJamesonSupplemental.pdf]

Supplementary Materials

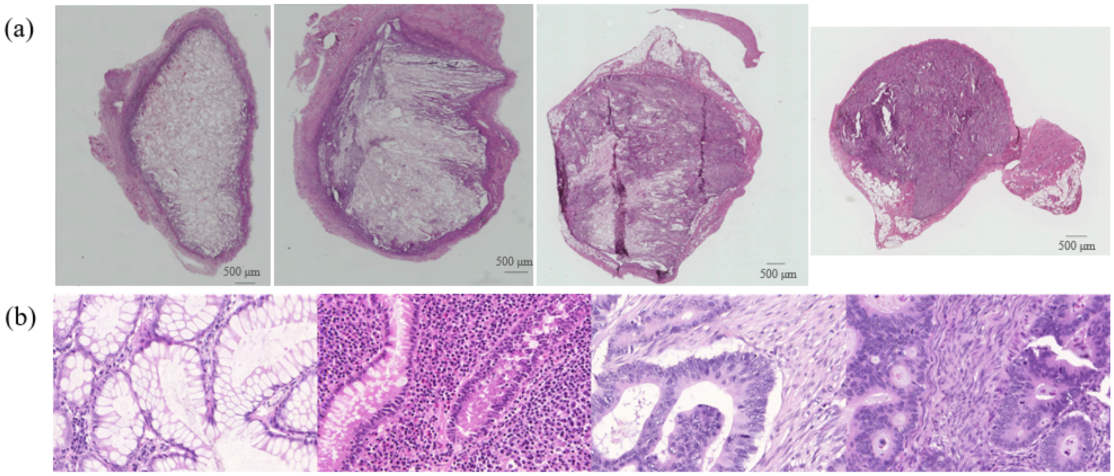

Figure S1: Example images from each histological dataset for adipose tissue (S1a) and cancer gland segmentation (S1b).

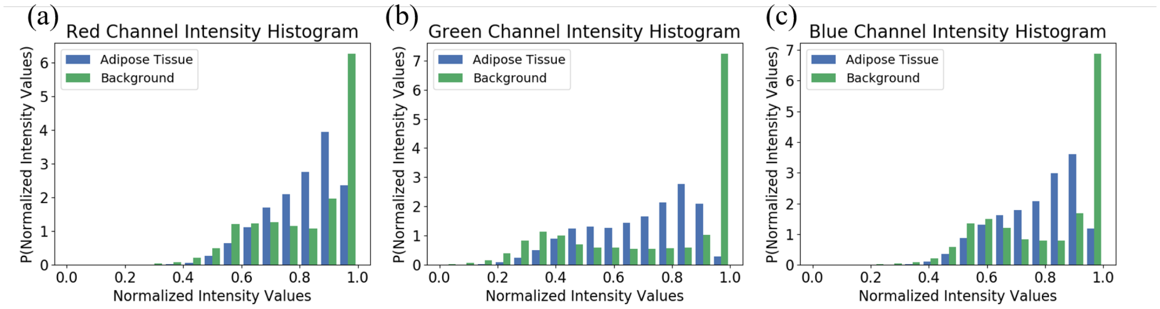

Figure S2: Color histograms (16 bins, equally spaced) of the normalized intensity values of each image in the SFBHI dataset (excludes test images).

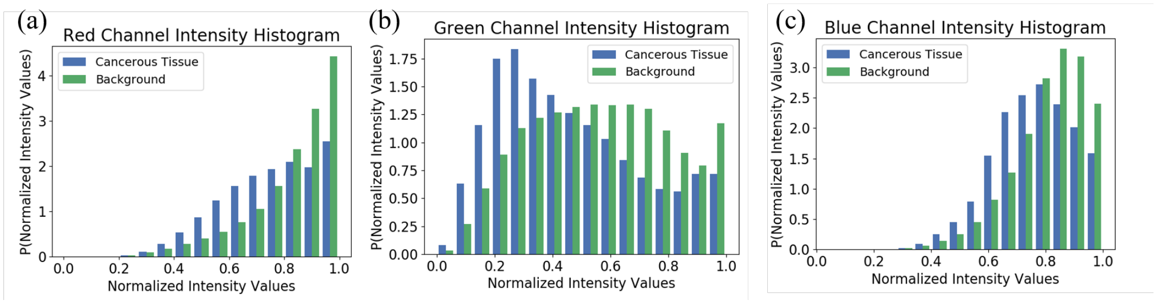

Figure S3: Color histograms (16 bins, equally spaced) of the normalized intensity values of each image in the GlaS dataset (excludes test images). We see here that the distribution of cancerous tissue pixels is similar to the distribution of background pixels (more overlap between the distributions than the SFBHI dataset).

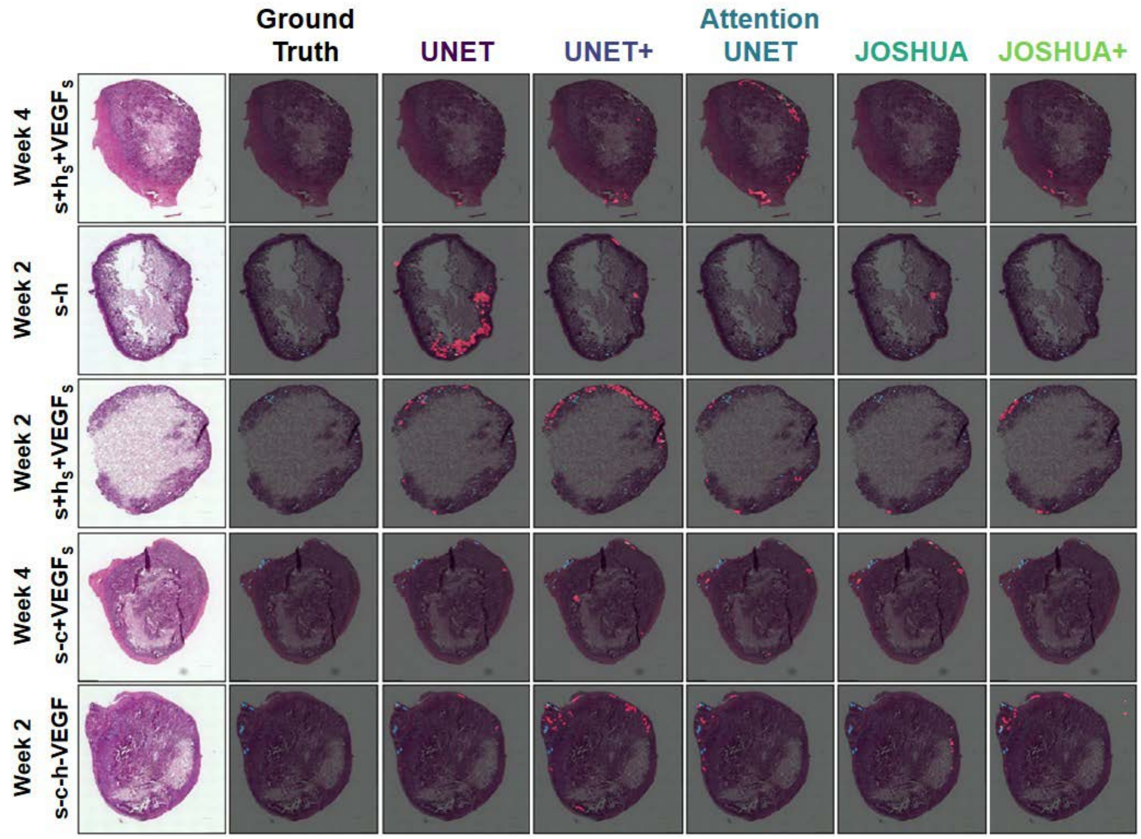

Figure S4: Example segmentation results on adipose-poor samples (*i.e.*, less than 1% of adipose pixels in image) from each model on the SFBHI dataset. The first column displays the input image and the corresponding ground truth label for the input image is shown in the second column. The remaining columns display the output from each model in comparison to the ground truth. Blue pixels correspond to the ground truth and red pixels correspond to the predicted output. Light green pixels indicate that both the ground truth and predicted output agree.

Table S1: Metrics for each data split on validation images in SFHBI dataset trained on UNET with binary cross entropy. The best average value is bolded. Data are shown as (mean ± SD.) One-way analysis of variance, followed by Dunnett's multiple-comparison test was computed. Asterisks [*] indicate significant differences as compared to validate on week 8 data split ($p < 0.05$). Validating on week 8 lead to the best performance but this is due to week 8 images having the most fat accumulation. This data split will be "easier" for the model but will not necessarily improve the robustness of the model to "harder" examples in practice (*i.e.*, little fat accumulation).

	Random	Stratified 5-fold (Time)	Stratified 5-fold (Condition)	4-fold with Time	Validate on week 8
Dice Coefficent	0.635±0.060*	0.0637±0.072*	0.638±0.092*	0.611±0.184*	**0.797±0.031**
IOU	0.748±0.038*	0.752±0.038*	0.751±0.051*	0.736±0.087*	**0.809±0.026**
Adipose Tissue IOU	0.521±0.069*	0.526±0.074*	0.526±0.095*	0.499±0.179*	**0.674±0.041**
Precision	0.689±0.069*	0.672±0.086*	0.686±0.087*	0.622±0.184*	**0.857±0.043**
Recall	0.613±0.066*	0.644±0.085*	0.631±0.105*	0.641±0.162*	**0.763±0.065**
Specificity	**0.992±0.003***	0.991±0.003*	**0.992±0.003***	0.986±0.011	0.980±0.006
Pixel Accuracy	97.720±0.787*	**97.860±0.606***	97.760±0.903*	97.480±1.235*	95.060±1.071

Table S2: Global distribution of random data split based on time.

			week			
			1	2	4	8
Fold	1	Training	26%	28%	25%	22%
		Validation	57%	17%	13%	13%
	2	Training	30%	28%	23%	19%
		Validation	38%	21%	21%	21%
	3	Training	35%	28%	19%	18%
		Validation	17%	22%	35%	26%
	4	Training	31%	27%	23%	19%
		Validation	35%	26%	17%	22%
	5	Training	36%	22%	21%	20%
		Validation	13%	43%	26%	17%
	all images		32%	26%	22%	20%

Table S3: Global distribution of random data split based on conditions.

			condition									
			silk	s+VEGFS	s+hS	s+hS+VEGFS	s-h	s-h+VEGFS	s-h-VEGF	s-c	s-c+VEGFS	s-c-h-VEGF
Fold	1	Training	16%	8%	6%	10%	9%	8%	11%	10%	12%	12%
		Validation	13%	4%	13%	13%	13%	8%	4%	17%	17%	0%
	2	Training	16%	5%	9%	8%	11%	10%	9%	13%	12%	9%
		Validation	13%	13%	4%	21%	4%	0%	13%	4%	17%	13%
	3	Training	14%	9%	9%	12%	7%	7%	10%	12%	14%	7%
		Validation	22%	0%	4%	4%	17%	9%	9%	9%	9%	17%
	4	Training	13%	7%	7%	12%	11%	9%	9%	10%	13%	11%
		Validation	26%	4%	9%	4%	4%	4%	13%	17%	13%	4%
	5	Training	18%	5%	7%	11%	10%	5%	10%	12%	14%	9%
		Validation	4%	13%	9%	9%	9%	17%	9%	9%	9%	13%
	all images		15%	7%	8%	10%	9%	8%	9%	11%	13%	9%

Table S4: Global distribution of stratified 5-fold time data split.

			week			
			1	2	4	8
Fold	1	Training	31%	27%	23%	19%
		Validation	33%	25%	21%	21%
	2	Training	31%	27%	23%	19%
		Validation	33%	25%	21%	21%
	3	Training	32%	27%	21%	20%
		Validation	30%	26%	26%	18%
	4	Training	32%	26%	22%	20%
		Validation	30%	30%	22%	18%
	5	Training	32%	27%	22%	19%
		Validation	30%	26%	22%	22%
	all images		32%	26%	22%	20%

Table S5: Global distribution of stratified 5-fold condition data split.

			condition									
			silk	s+VEGFS	s+hS	s+hS+VEGFS	s-h	s-h+VEGFS	s-h-VEGF	s-c	s-c+VEGFS	s-c-h-VEGF
Fold	1	Training	15%	6%	8%	11%	10%	9%	10%	11%	13%	9%
		Validation	17%	8%	8%	8%	8%	4%	8%	13%	13%	13%
	2	Training	15%	6%	8%	11%	10%	8%	10%	11%	13%	10%
		Validation	17%	8%	8%	8%	8%	8%	8%	13%	13%	8%
	3	Training	15%	7%	9%	11%	9%	7%	10%	11%	13%	10%
		Validation	17%	4%	4%	9%	13%	9%	9%	13%	13%	9%
	4	Training	16%	7%	7%	10%	10%	7%	9%	12%	13%	10%
		Validation	13%	4%	9%	13%	9%	9%	13%	9%	13%	9%
	5	Training	16%	6%	7%	10%	10%	7%	10%	12%	13%	10%
		Validation	13%	9%	9%	13%	9%	9%	9%	9%	13%	9%
	all images		15%	7%	8%	10%	9%	8%	9%	11%	13%	9%

Table S6: Global distribution of 4-fold with time data split.

			week			
			1	2	4	8
Fold	1	Training	X	X	X	
		Validation				X
	2	Training		X	X	X
		Validation	X			
	3	Training	X		X	X
		Validation		X		
	4	Training	X	X		X
		Validation			X	

Table S7: Global distribution of validate on week 8 data split.

			week			
			1	2	4	8
Fold	1	Training	X	X	X	
		Validation				X
	2	Training	X	X	X	
		Validation				X
	3	Training	X	X	X	
		Validation				X
	4	Training	X	X	X	
		Validation				X

Table S8: Metrics for each model on images in SFHBI dataset. The best average value is bolded. Data are shown as (mean ± SD.) One-way analysis of variance, followed by Dunnett's multiple-comparison test was computed. Asterisks [*] indicate significant differences as compared to UNET ($p < 0.05$). All of our proposed models (UNET+, JOSHUA, and JOSHUA+) improve results in comparison the the baseline UNET and Attention UNET. The statistical texture features and new fusion approach effectively identified the adipose tissue in our SFBHI dataset.

	UNET	UNET+	Attention UNET	JOSHUA	JOSHUA+
Dice Coefficient	0.635±0.060	0.663±0.049	0.659±0.045	0.647±0.042	**0.674±0.026**
IOU	0.748±0.038	0.767±0.028	0.764±0.025	0.757±0.025	**0.774±0.017***
Adipose Tissue IOU	0.521±0.069	0.554±0.054	0.549±0.048	0.535±0.049	**0.567±0.033**
Precision	0.689±0.069	0.703±0.060	0.698±0.042	0.684±0.059	**0.704±0.038**
Recall	0.613±0.066	0.654±0.051	0.655±0.055	0.654±0.056	**0.675±0.032***
Specificity	0.992±0.003	**0.993±0.002**	0.992±0.002	0.992±0.003	**0.993±0.002**
Pixel Accuracy	97.72±0.787	98.13±0.320	98.06±0.390	97.98±0.233	**98.27±0.304***

Table S9: Metrics for each model on images in GlaS dataset trained with binary cross entropy loss. The best average value is bolded. Data are shown as (mean ± SD.) One-way analysis of variance, followed by Dunnett's multiple-comparison test was computed. Asterisks [*] indicate significant differences as compared to UNET ($p < 0.05$). For most metrics, JOSHUA improves performance showing the value of statistical texture features for cancer identification.

	UNET	UNET+	Attention UNET	JOSHUA	JOSHUA+
Dice Coefficient	0.889±0.038	0.895±0.012	0.887±0.022	**0.897±0.011**	0.883±0.020
IOU	0.797±0.072	0.810±0.020	0.799±0.030	**0.812±0.015**	0.795±0.025
Cancerous Glands IOU	0.812±0.051	0.821±0.015	0.810±0.029	**0.823±0.015**	0.804±0.027
Precision	**0.922±0.019**	0.922±0.021	0.907±0.037	0.920±0.026	0.905±0.035
Recall	0.875±0.067	0.883±0.028	0.885±0.024	**0.888±0.023**	0.880±0.029
Specificity	**0.908±0.012**	0.908±0.018	0.895±0.035	0.907±0.021	0.893±0.025
Pixel Accuracy	88.96±4.98	89.67±1.38	88.97±2.19	**89.87±1.07**	88.80±1.73
