# Supplementary figures and images for: Jointly Optimized Spatial Histogram UNET Architecture (JOSHUA) for Adipose Tissue Segmentation
Source: BME Front. 2022 Jun 3;2022:9854084. doi: 10.34133/2022/9854084 (PMC10521712; doi:10.34133/2022/9854084)

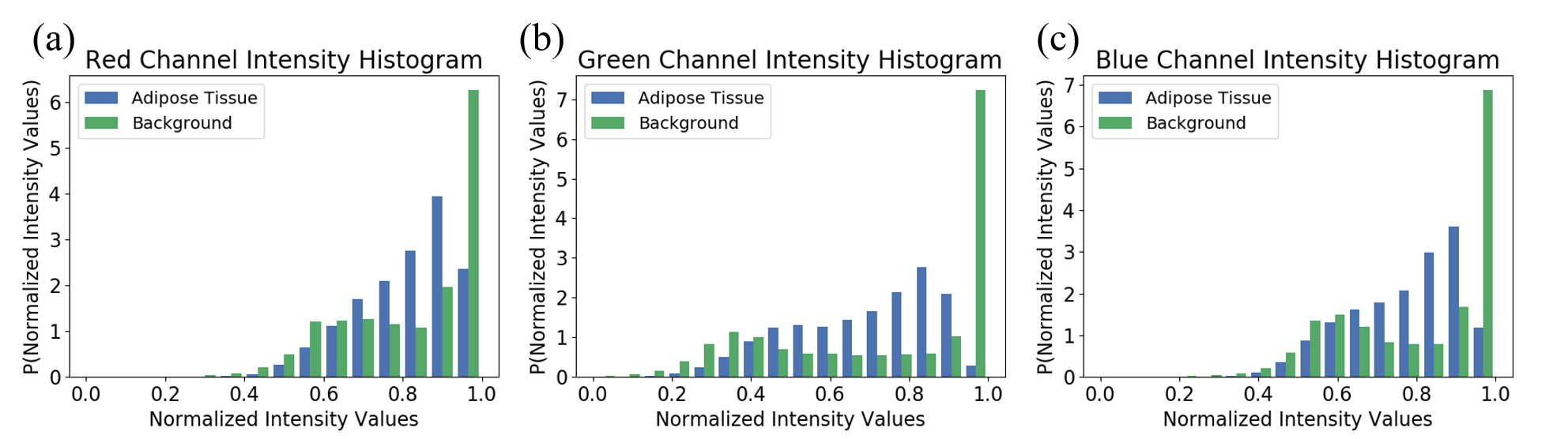

Supplement: Supplementary Materials — Figure S1: example images from each histological dataset for adipose tissue (S1a) and cancer gland segmentation (S1b). Figure S2: color histograms (16 bins, equally spaced) of the normalized intensity values of each image in the SFBHI dataset (excludes test images). Figure S3: color histograms (16 bins, equally spaced) of the normalized intensity values of each image in the GlaS dataset (excludes test images). Figure S4: example segmentation results on adipose-poor samples (i.e., less than 1% of adipose pixels in image) from each model on the SFBHI dataset. Table S1: metrics for each data split on validation images in SFHBI dataset trained on UNET with binary cross entropy. Table S2: global distribution of random data split based on time. Table S3: global distribution of random data split based on conditions. Table S4: global distribution of stratified 5-fold time data split. Table S5: global distribution of stratified 5-fold condition data split. Table S6: global distribution of 4-fold with time data split. Table S7: global distribution of validate on week 8 data split. Table S8: metrics for each model on images in SFHBI dataset Table S9: metrics for each model on images in GlaS dataset trained with binary cross entropy loss. [file 9854084.f1.zip › FigureS2.png]

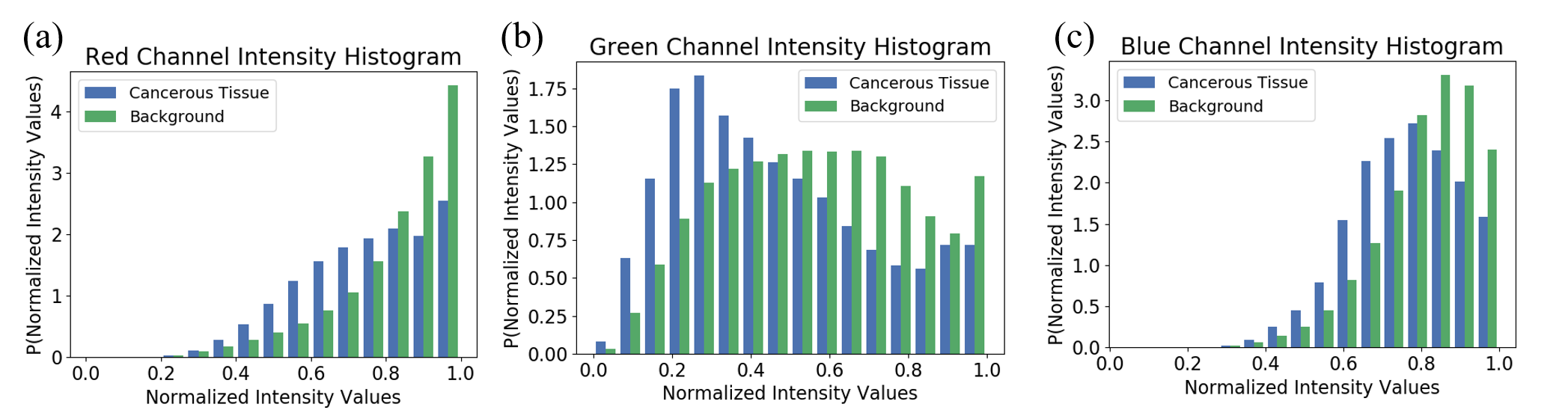

Supplement: Supplementary Materials — Figure S1: example images from each histological dataset for adipose tissue (S1a) and cancer gland segmentation (S1b). Figure S2: color histograms (16 bins, equally spaced) of the normalized intensity values of each image in the SFBHI dataset (excludes test images). Figure S3: color histograms (16 bins, equally spaced) of the normalized intensity values of each image in the GlaS dataset (excludes test images). Figure S4: example segmentation results on adipose-poor samples (i.e., less than 1% of adipose pixels in image) from each model on the SFBHI dataset. Table S1: metrics for each data split on validation images in SFHBI dataset trained on UNET with binary cross entropy. Table S2: global distribution of random data split based on time. Table S3: global distribution of random data split based on conditions. Table S4: global distribution of stratified 5-fold time data split. Table S5: global distribution of stratified 5-fold condition data split. Table S6: global distribution of 4-fold with time data split. Table S7: global distribution of validate on week 8 data split. Table S8: metrics for each model on images in SFHBI dataset Table S9: metrics for each model on images in GlaS dataset trained with binary cross entropy loss. [file 9854084.f1.zip › FigureS3.png]

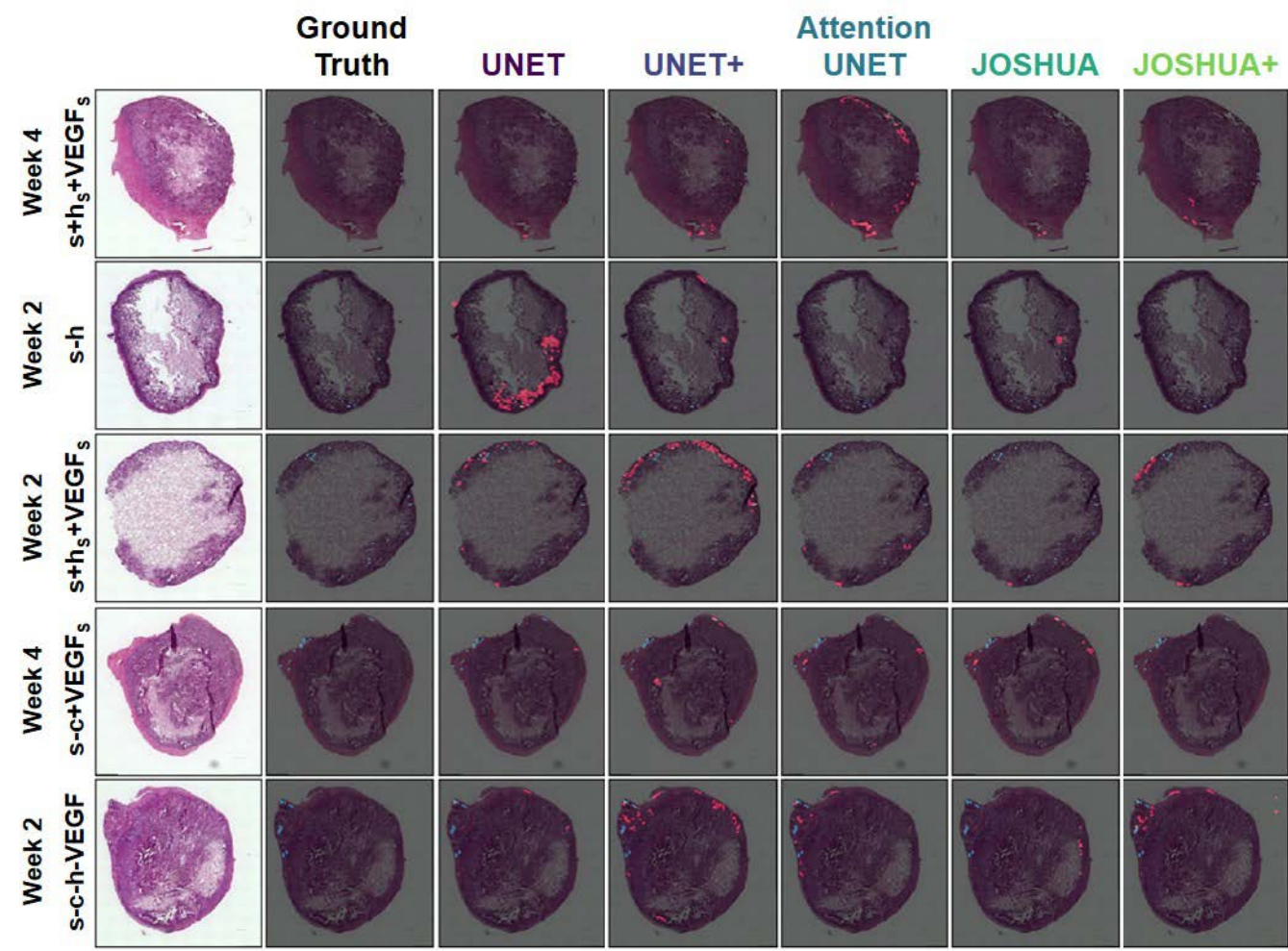

Supplement: Supplementary Materials — Figure S1: example images from each histological dataset for adipose tissue (S1a) and cancer gland segmentation (S1b). Figure S2: color histograms (16 bins, equally spaced) of the normalized intensity values of each image in the SFBHI dataset (excludes test images). Figure S3: color histograms (16 bins, equally spaced) of the normalized intensity values of each image in the GlaS dataset (excludes test images). Figure S4: example segmentation results on adipose-poor samples (i.e., less than 1% of adipose pixels in image) from each model on the SFBHI dataset. Table S1: metrics for each data split on validation images in SFHBI dataset trained on UNET with binary cross entropy. Table S2: global distribution of random data split based on time. Table S3: global distribution of random data split based on conditions. Table S4: global distribution of stratified 5-fold time data split. Table S5: global distribution of stratified 5-fold condition data split. Table S6: global distribution of 4-fold with time data split. Table S7: global distribution of validate on week 8 data split. Table S8: metrics for each model on images in SFHBI dataset Table S9: metrics for each model on images in GlaS dataset trained with binary cross entropy loss. [file 9854084.f1.zip › FigureS4.pdf]
